# Preoperative low C-reactive protein–albumin–lymphocyte (CALLY) index is a poor prognostic indicator for overall survival in patients undergoing surgery for pancreatic ductal adenocarcinoma

**DOI:** 10.1007/s00595-026-03270-8

**Published:** 2026-03-19

**Authors:** Yu Matsumoto, Yuichiro Otsuka, Hiroka Hosaka, Yoji Kajiwara, Rei Okada, Yuko Ito, Masaru Tsuchiya, Hideaki Shimada

**Affiliations:** 1 Department of Surgery, Toho University School of Medicine, 6-11-1 Omori-Nishi, Ota-ku, Tokyo, 143-8541 Japan; 2 Department of Surgery, Toho University Sakura Medical Center, Chiba, Japan; 3 Health Management Center, Japan Community Health Care Organization (JCHO) Funabashi Central Hospital, Chiba, Japan

**Keywords:** Pancreatic cancer, Surgery, Overall survival, CALLY index, Biomarker

## Abstract

**Purpose:**

This study aimed to investigate the prognostic impact of preoperative indicators that reflect systemic inflammatory, nutritional, and immune status, focusing on the C-reactive protein–albumin–lymphocyte (CALLY) index in patients undergoing surgery for pancreatic ductal adenocarcinoma.

**Methods:**

This retrospective study included 120 patients (stage I = 19, II = 94, III = 7) who underwent surgical resection for pancreatic ductal adenocarcinoma between 2007 and 2019. Preoperative laboratory data and the CALLY index were collected to evaluate their impact on overall survival (OS). A receiver operating characteristic (ROC) curve analysis was used to identify the optimal cutoff values.

**Results:**

The optimal cutoff value for the preoperative CALLY index was 4.0. Patients with a low CALLY index (< 4.0) demonstrated a significantly worse OS than those with a high CALLY index (≥ 4.0) (*P* = 0.001). A multivariate analysis of composite indices identified low CALLY index (*P* = 0.001) as an independent prognostic factor, along with positive lymph node metastasis (*P* = 0.035) and the absence of adjuvant chemotherapy (*P* = 0.005).

**Conclusion:**

The CALLY index reflects the preoperative systemic inflammatory, nutritional, and immune status. It is a convenient and practical prognostic predictor of OS in patients undergoing surgery for pancreatic ductal adenocarcinoma.

**Supplementary Information:**

The online version contains supplementary material available at 10.1007/s00595-026-03270-8.

## Introduction

 Pancreatic cancer has a poor prognosis, and its incidence and mortality rates are increasing globally [1]. Surgery is the only curative treatment for pancreatic cancer; however, the five-year survival rate is only 15–25%, even among patients who undergo surgery [2]. In particular, the incidence of pancreatic ductal adenocarcinoma (PDAC), which accounts for the majority of pancreatic cancers, is on the rise in Japan. It is now the fourth leading cause of cancer-related deaths after lung cancer, colorectal cancer, and gastric cancer [3]. Therefore, the development of simple and reliable biomarkers that accurately predict preoperative patient outcomes is crucial for identifying high-risk patients and planning treatment strategies.

Clinical and pathological factors, including the tumor–lymph node–metastasis staging system and tumor markers such as carbohydrate antigen (CA) 19 − 9 and carcinoembryonic antigen (CEA), are traditionally used to assess the prognosis of pancreatic cancer [4]. The association between systemic inflammation, nutritional status, and immune status in pancreatic cancer, as reflected by C-reactive protein (CRP), albumin, neutrophils, lymphocytes, and platelets, has attracted increasing interest [5]. Furthermore, composite prognostic indices, including the CRP–albumin–lymphocyte (CALLY) index [6], systemic inflammation index (SII) [7], neutrophil-to-lymphocyte ratio (NLR) [8], and predicted nutritional index (PNI) [9] have been developed, indicating their potential usefulness as prognostic markers. In particular, the CALLY index is a newer indicator that has recently been useful for gastroenterological cancers, including esophageal and gastric cancer [10–23]. However, only three studies have reported pancreatic cancer [6, 24, 25], and comprehensive comparative studies of other major composite indices within a single model are rare.

Therefore, this study aimed to investigate the usefulness of the CALLY index—a composite index reflecting systemic inflammatory, nutritional, and immune status—by comparing it with similar indices to identify its effectiveness for predicting prognosis in patients undergoing surgery for PDAC.

## Methods

### Patients

**Fig. 1:**
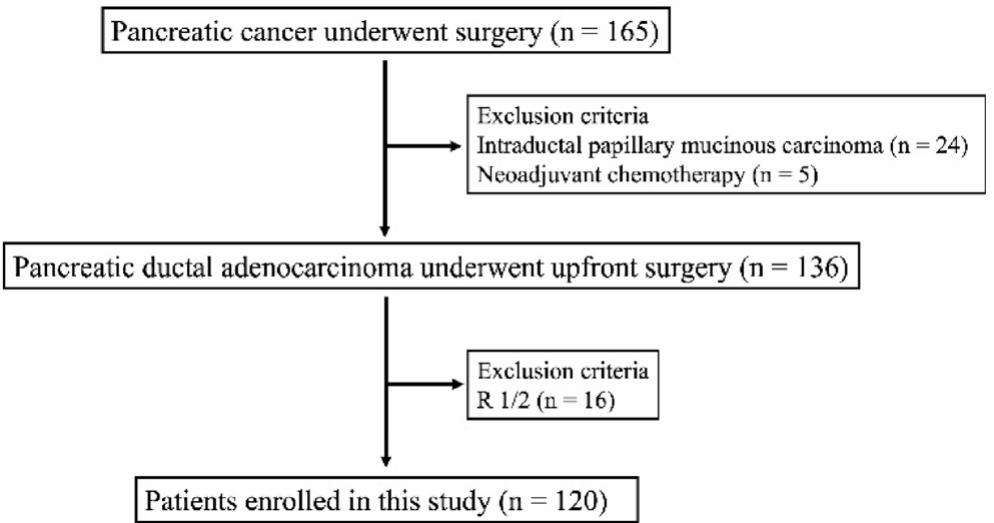
Flowchart of patient selection for this study

This retrospective study included 120 patients (stage I = 19, II = 94, and III = 7) with a median age of 71 years (range, 33–85 years) who were diagnosed with PDAC between 2007 and 2019 at Toho University Omori Medical Center. All patients were preoperatively diagnosed with resectable disease and underwent upfront surgery with curative R0 resection (Fig. 1). Overall survival (OS) was defined as the interval from the date of surgery to the date of death or last follow-up. The Ethics Committee of Toho University Omori Medical Center approved the present study (#M24224 M23174 21320 21039 20200 20196 19056 18002), which adhered to the Declaration of Helsinki. Information about the study was disclosed on the institution’s website and potential participants were free to opt out at any time.

### Measurement of clinicopathological factors

Blood sampling data, including clinicopathological factors, were collected within seven days preoperatively. The eighth edition of the Japanese Classification of Pancreatic Cancer was used to identify pathological findings based on the tumor–node–metastasis classification [26].

### Measurement of the CALLY index and composite indices

The CALLY index was defined as the serum albumin level multiplied by the peripheral lymphocyte count and divided by the serum CRP level multiplied by 10^4^ ([albumin × lymphocyte]/[CRP × 10^4^]). The SII is defined as the platelet count multiplied by the peripheral neutrophil count divided by the peripheral lymphocyte count ([platelet × neutrophil]/lymphocyte). PNI was defined as the sum of the serum albumin level multiplied by 10 and the peripheral lymphocyte count multiplied by 0.005 ([albumin × 10] + [lymphocyte × 0.005]). The NLR was defined as the ratio of peripheral neutrophil count to peripheral lymphocyte count (neutrophil-to-lymphocyte ratio).

### Statistical analysis

The cutoff value of the biomarker was identified according to receiver operating characteristic (ROC) analysis based on the survival status at the 5-year follow-up. Continuous variables were expressed as medians and ranges. Categorical variables were presented as numbers and percentages. Mann–Whitney U test and Fisher’s exact test were used to compare continuous and dichotomous variables, respectively. The Kaplan–Meier method was employed to estimate OS, and the log-rank test was performed to assess between-group differences. A Cox proportional hazards regression analysis was used for multivariate analyses. EZR was used for all statistical analyses [27]. A two-sided P-value < 0.05 indicated statistical significance.

## Results

### Patient characteristics

This study included 120 patients with histologically confirmed PDAC who were preoperatively diagnosed with resectable disease and underwent upfront curative R0 resection. Table 1 summarizes the detailed clinicopathological characteristics of the present study. The median age of the patients was 71 years (range, 33–85 years), and the median body mass index was 21.9 kg/m² (range, 16–32 kg/m²). The study population comprised of 61 women (51%) and 59 men (49%). Preoperative obstructive jaundice drainage was performed in 42 (35%) patients. Pancreaticoduodenectomy performed in 67 patients (55%) was the most common surgical procedure, followed by distal pancreatectomy in 38 patients (32%) and total pancreatectomy in 15 patients (13%). Regarding pathological findings, 99 patients (83%) had advanced T-stage tumors (T3–T4), whereas 40 (33%) had lymph node metastasis (N1). Major postoperative complications (Clavien–Dindo grade ≥ III) occurred in 32 patients (27%). Most patients (*n* = 96, 80%) received adjuvant chemotherapy. The median preoperative serum levels of CA 19 − 9 were 76 U/mL (range, 0.6–10690), and the median CEA levels were 2.9 ng/mL (range, 0.6–105) Table 2.

**Table 1:** Patients’ characteristics (*n* = 120, MST = 29.4months )

Variables		*n* = 120 median (range) or n (%)
**Age**,** years**		71 (33-85)
**Body mass index**,** kg/m**^**2**^		21.9 (16-32)
**Sex**	Female	61 (51%)
	Male	59 (49%)
**Obstructive jaundice drainage**	Yes	42 (35%)
	No	78 (65%)
**Surgical procedure**	TP	15 (13%)
	PD	67 (55%)
	DP	38 (32%)
**Postoperative complications**	C-D <Ⅲ	88 (73%)
	C–D ≥Ⅲ	32 (27%)
**Pathological T factor**	T1T2	21 (17%)
	T3T4	99 (83%)
**Pathological N factor**	N0	80 (67%)
	N1	40 (33%)
**Adjuvant chemotherapy**	Yes	96 (80%)
	No	24 (20%)
**CA 19-9**,** U/mL**		76 (0.6-10690)
**CEA**,** ng/mL**		2.9 (0.6-105)

*MST* Median survival time, *TP* total pancreatectomy, *PD* pancreaticoduodenectomy, *DP* distal pancreatectomy,

*C–D* Clavien–Dindo grade, *CA19-9* Carbohydrate antigen 19 − 9,

*CEA* Carcinoembryonic antigen

**Table 2:** Comparison of clinicopathological factors between the low and high CALLY index groups

Variables		Low CALLY index <4.0 (*n* = 56)	High CALLY index ≥4.0 (*n* = 64)	*p*-value^a^
		Median (range) or numbers		
**Age (years)**		64 (33-85)	63.5 (46-84)	0.645
**Body mass index (kg/m** ^**2**^ **)**		21.9 (15.5-32.2)	22 (15.9-31.3)	0.264
**Sex**	Female / Male	31 / 25	30 / 34	0.637
**Obstructive jaundice drainage**	Yes / No	24 / 32	18 / 46	0.125
**Surgical procedure**	TP or PD / DP	40 / 16	42 / 22	0.558
**Postoperative complications**	C–D ≥Ⅲ / C-D <Ⅲ	17 / 39	15 / 49	0.415
**Pathological T factor**	T3T4 / T1T2	49 / 7	50 / 14	0.231
**Pathological N factor**	N1 / N0	20 / 36	20 / 44	0.699
**Adjuvant chemotherapy**	No / Yes	13 / 43	Nov-53	0.495
**CA 19-9 (U/mL)**		105.5 (2-10690)	48.6 (0.6-2079)	**0.016**
**CEA (ng/mL)**		3 (0.7-105.3)	2.8 (0.6-23.8)	0.764
**Neutrophil (/μL)**		3099 (1196-8841)	3134 (1542-8336)	0.966
**Lymphocyte (/μL)**		1307 (529-2464)	1678 (1040-3164)	**<0.001**
**CRP (mg/dL)**		0.3 (0.1-4.6)	0.1 (0.1-0.2)	**<0.001**
**Albumin (g/dL)**		3.7 (2.6-4.4)	4 (3-4.7)	**<0.001**
**SII (×10** ^**3**^ **)**		528 (142-2287)	437 (110-1579)	**0.025**
**PNI**		43.3 (30.5-54.7)	48.7 (40-54.2)	**<0.001**
**NLR**		2.4 (0.9-9.9)	2 (0.6-7.2)	**0.003**

^a^ Mann-Whitney U-test or Fisher’s exact probability test

*CALLY index* C-reactive protein–albumin–lymphocyte index, *TP* total pancreatectomy, *PD* pancreaticoduodenectomy, *DP* distal pancreatectomy, *C–D* Clavien–Dindo grade, *CA19-9* carbohydrate antigen 19 − 9, *CEA* carcinoembryonic antigen, *SII* systemic inflammation index, *PNI* prognostic nutritional index, *NLR* neutrophil-to-lymphocyte ratio

### The cutoff value of the composite indices

**Fig. 2:**
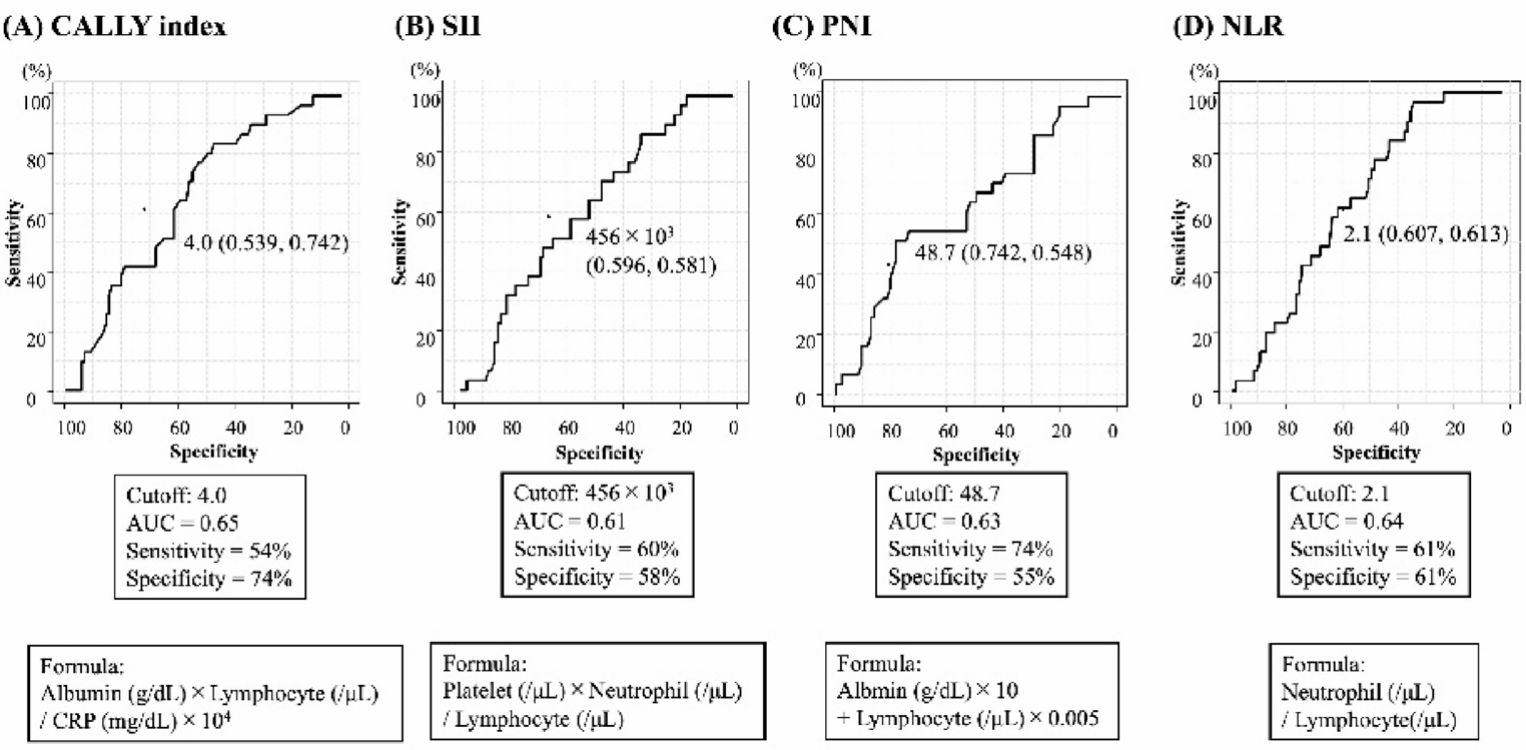
The area under the receiver operating characteristic curve used to determine the cutoff values for CALLY index, SII, PNI, and NLR in predicting the 5-year overall survival. *AUC* Area under the curve, *CALLY index* C-reactive protein–albumin–lymphocyte index, *SII* systemic inflammation index, *PNI* prognostic nutritional index, *NLR* neutrophil-to-lymphocyte ratio, *CRP* C-reactive protein

An ROC curve analysis was conducted to identify the optimal cutoff values of the preoperative composite indices for predicting OS. The results are shown in Fig. 2. The cutoff values were 4.0 for the CALLY index (AUC = 0.65, sensitivity = 54%, specificity = 74%; Fig. 2A), 456 × 10³ for the SII (AUC = 0.61, sensitivity = 60%, specificity = 58%; Fig. 2B), 48.7 for the PNI (AUC = 0.63, sensitivity = 74%, specificity = 55%; Fig. 2C), and 2.1 for the NLR (AUC = 0.64, sensitivity = 61%, specificity = 61%; Fig. 2D).

### Comparison of clinicopathological factors between the low and high CALLY index groups

Patients were categorized based on the optimal cutoff value of the CALLY index into a low CALLY index group (< 4.0, *n* = 56) and a high CALLY index group (≥ 4.0, *n* = 64). The clinicopathological characteristics of these two groups were compared, and Table 2 summarizes the results. No significant differences in age, body mass index, sex, surgical procedure, postoperative complications, pathological T factor, or pathological N factor were observed between the two groups. However, the low CALLY index group demonstrated a significantly higher CA 19 − 9 level than the high CALLY index group (*P* = 0.016). Consistent with the formula for the CALLY index, the low CALLY group showed significantly lower lymphocyte counts (*P* < 0.001), higher CRP levels (*P* < 0.001), and lower albumin levels (*P* < 0.001). Furthermore, the low CALLY group had significantly higher SII (*P* = 0.025), lower PNI (*P* < 0.001), and higher NLR (*P* = 0.003).

### Survival analysis based on composite indices

**Fig. 3:**
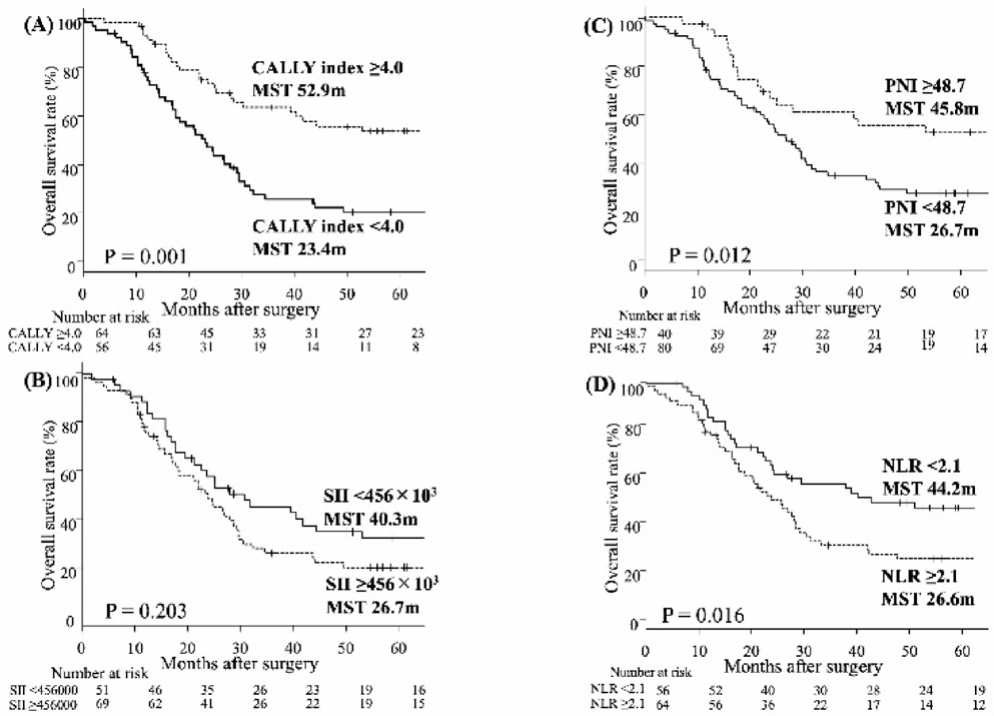
Comparison of the overall survival between the high and low groups for CALLY index, SII, PNI, and NLR. *CALLY index* C-reactive protein–albumin–lymphocyte index, *SII* systemic inflammation index, *PNI* prognostic nutritional index, *NLR* neutrophil-to-lymphocyte ratio, *MST* median survival time

The Kaplan–Meier method and log-rank test were used to compare the OS of patients grouped based on the cutoff values for each composite index. Figure 3 illustrates the survival curves. The low CALLY index group (< 4.0) was significantly associated with a worse OS than the high CALLY index group (≥ 4.0) (*P* = 0.001, Fig. 3A). No significant difference in OS was found between the high SII group (≥ 456 × 10³) and low SII group (< 456 × 10³) (*P* = 0.203, Fig. 3B). The low PNI group (< 48.7) was significantly associated with worse OS than the high PNI group (≥ 48.7) (*P* = 0.012; Fig. 3C). Finally, the high NLR group (≥ 2.1) was significantly associated with a worse OS than the low NLR group (< 2.1) (*P* = 0.016; Fig. 3D).

### Prognostic factor analysis, including composite indices

Univariate (log-rank test) and multivariate (Cox regression) analyses were performed to evaluate the prognostic value of the composite indices. Table 3 summarizes the results of the univariate analysis. A univariate analysis identified that positive lymph node metastasis (*P* = 0.020), no adjuvant chemotherapy (*P* = 0.018), high CA 19 − 9 level (≥ 40 U/mL) (*P* = 0.006), high CEA level (≥ 5 ng/mL) (*P* = 0.027), low CALLY index (< 4.0) (*P* = 0.001), low PNI (< 48.7) (*P* = 0.012), and high NLR (≥ 2.1) (*P* = 0.016) were significantly associated with a poor OS.

**Table 3:** A univariate analysis of the clinicopathological factors predicting overall survival, including composite indices

Variables		Number of patients (*n* = 120)	*p*-value^a^
**Age (years)**	≥70 / <70	69 / 51	0.506
**Body mass index (kg/m** ^**2**^ **)**	≥22 / <22	59 / 61	0.301
**Sex**	Female / Male	61 / 59	0.682
**Obstructive jaundice drainage**	Yes / No	42 / 78	0.088
**Surgical procedure**	TP or PD / DP	82 / 38	0.508
**Postoperative complications**	C–D ≥Ⅲ / C-D <Ⅲ	32 / 88	0.558
**Pathological T factor**	T3T4 / T1T2	99 / 21	0.443
**Pathological N factor**	N1 / N0	40 / 80	**0.020**
**Adjuvant chemotherapy**	No / Yes	24 / 96	**0.018**
**CA 19-9 (U/mL)**	≥40 / <40	68 / 52	**0.006**
**CEA (ng/mL)**	≥5 / <5	29 / 91	**0.027**
**CALLY index**	**<4.0 / ≥4.0**	**56 / 64**	**0.001**
**SII (×10** ^**3**^ **)**	≥456 / <456	69 / 51	0.203
**PNI**	<48.7 / ≥48.7	80 / 40	**0.012**
**NLR**	≥2.1 / <2.1	64 / 56	**0.016**

^a^Log-rank test

*TP* total pancreatectomy, *PD* pancreaticoduodenectomy, *DP* distal pancreatectomy, *C–D* Clavien–Dindo grade, *CA19-9* carbohydrate antigen 19 − 9, *CEA* carcinoembryonic antigen, *CALLY index* C-reactive protein–albumin–lymphocyte index, *SII* systemic inflammation index, *PNI* prognostic nutritional index, *NLR* neutrophil-to-lymphocyte ratio

To avoid multicollinearity, each composite index was evaluated by using a separate multivariate model (Table 4). In Model 1, which included the CALLY index along with other significant clinicopathological factors, a low CALLY index was identified as an independent predictor of a poor OS (hazard ratio [HR] = 2.23, 95% confidence interval [CI] = 1.39–3.59, *P* = 0.001), along with positive lymph node metastasis (HR = 1.75, 95% CI = 1.04–2.95, *P* = 0.035) and no adjuvant chemotherapy (HR = 2.31, 95% CI = 1.28–4.16, *P* = 0.005). In this model, high CA 19 − 9 levels did not reach statistical significance (*P* = 0.056). In Model 2, which included PNI along with other significant clinicopathological factors, low PNI was identified as an independent predictor of a poor OS (HR = 1.87, 95% CI = 1.09–3.21, *P* = 0.024), along with positive lymph node metastasis (HR = 1.76, 95% CI = 1.05–2.95, *P* = 0.033), no adjuvant chemotherapy (HR = 2.04, 95% CI = 1.13–3.68, *P* = 0.017), and high CA 19 − 9 level (HR = 1.76, 95% CI = 1.07–2.89, *P* = 0.025). In Model 3, which included NLR along with other significant clinicopathological factors, high NLR was identified as an independent predictor of a poor OS (HR = 2.21, 95% CI = 1.31–3.74, *P* = 0.003), along with positive lymph node metastasis (HR = 1.77, 95% CI = 1.06–2.95, *P* = 0.029), no adjuvant chemotherapy (HR = 2.29, 95% CI = 1.28–4.09, *P* = 0.005), and high CA 19 − 9 level (HR = 2.16, 95% CI = 1.28–3.65, *P* = 0.004).

**Table 4:** A multivariate analysis of the clinicopathological factors predicting overall survival, including composite indices

Variables		Model 1 (with CALLY index)		Model 2 (with PNI)		Model 3 (with NLR)	
		HR (95% CI)	*p*-value^a^	HR (95% CI)	*p*-value^a^	HR (95% CI)	*p*-value^a^
**Pathological N factor**	N1 / N0	1.75 (1.04-2.95)	**0.035**	1.76 (1.05-2.95)	**0.033**	1.77 (1.06-2.95)	**0.029**
**Adjuvant chemotherapy**	No / Yes	2.31 (1.28-4.16)	**0.005**	2.04 (1.13-3.68)	**0.017**	2.29 (1.28-4.09)	**0.005**
**CA 19-9 (U/mL)**	≥40 / <40	1.62 (0.99-2.67)	0.056	1.76 (1.07-2.89)	**0.025**	2.16 (1.28-3.65)	**0.004**
**CEA (ng/mL)**	≥5 / <5	1.46 (0.86-2.49)	0.159	1.35 (0.79-2.27)	0.264	1.07 (0.62-1.85)	0.264
**CALLY index**	<4.0 / ≥4.0	2.23 (1.39-3.59)	**0.001**				
**PNI**	<48.7 / ≥48.7			1.87 (1.09-3.21)	**0.024**		
**NLR**	≥2.1 / <2.1					2.21 (1.31-3.74)	**0.003**

^a^Cox Regression Analysis

*HR* hazard ratio, *CI* confidence interval, *CA19-9* Carbohydrate antigen 19 − 9, *CEA* Carcinoembryonic antigen, *CALLY index* C-reactive protein–albumin–lymphocyte index, *PNI* Prognostic nutritional index, *NLR* Neutrophil-to-lymphocyte ratio

## Discussion

In this study, we investigated the usefulness of the CALLY index, a composite index reflecting systemic inflammatory, nutritional, and immune status, by comparing it with similar indices to identify its effectiveness in predicting prognosis in patients undergoing surgery for PDAC. We found that the CALLY index was an independent predictor of a poor OS.

The biological rationale for the prognostic ability of the CALLY index is its comprehensive components. For example, NLR, which was also an independent prognostic factor in this study, serves as a powerful indicator of the inflammatory and immune status of the host through the presence of neutrophils and lymphocytes [7]. Meanwhile, the CALLY index assesses inflammation using CRP, an acute-phase marker, to evaluate the nutritional status—a key factor affecting the prognosis of patients with cancer—using albumin and measures immune status using lymphocytes. Therefore, the uniqueness and superiority of the CALLY index lie in its ability to simultaneously measure three interrelated biological factors: inflammation, nutrition, and immunity. Furthermore, these three biomarkers are part of routine preoperative blood tests and their calculation is simple. They involve no special costs or procedures; thus, their high practicality makes them easy to implement in clinical settings. This is a significant advantage of this indicator. This study initially focused on individual factors such as neutrophil count, lymphocyte count, and albumin levels (Supplemental Fig. 1, Supplemental Fig. 2). The addition of CRP, which reflects inflammation, was expected to improve the accuracy of the CALLY index compared to the PNI as a conventional indicator; however, it did not significantly improve the AUC. When interpreting these results, several biological limitations should be acknowledged. PDAC is widely recognized as a ‘cold tumor’ characterized by an ‘immune-desert’ microenvironment and T-cell exclusion [28]. These unique immunological features may weaken the correlation between peripheral lymphocyte counts and intratumoral immune activity compared with other gastrointestinal cancers. Consequently, the prognostic impact and predictive superiority of systemic immune-based markers, including the CALLY index, may be more constrained in PDAC.

A notable finding of this study was that the low CALLY index group demonstrated significantly higher CA 19 − 9 levels. CA 19 − 9 is widely recognized as a marker of tumor activity and burden [29]. This association indicates that the CALLY index may not only assess the systemic condition of the host but also indirectly reflect the underlying biological malignancy of the tumor. CA19-9 is the gold standard marker for gastrointestinal cancers; however, its usefulness is limited in patients lacking the Lewis antigen. In contrast, the CALLY index is a simple marker applicable to all cases. Furthermore, while CA19-9 reflects tumor-related factors, the CALLY index reflects host-related factors and the host reserve capacity. For patients with PDAC, evaluating the systemic status using the CALLY index in conjunction with CA19-9 is clinically important as it may help predict the tolerability and feasibility of continuing adjuvant chemotherapy. Although a subgroup analysis limited to CA 19-9-positive cases (*n* = 68) showed less definitive results than the total cohort (Supplemental Fig. 3, Supplemental Tables 1 and 2), we consider it clinically essential to evaluate all PDAC cases using the CALLY index to provide a comprehensive assessment of the host’s systemic state. In this study, the ROC AUC for the CALLY index in predicting 5-year survival was 0.65, and the hazard ratio was 2.23. These values indicate that the CALLY index is a moderate prognostic predictor. Therefore, the CALLY index is considered useful not as a standalone diagnostic marker but rather as an auxiliary tool for evaluating a patient’s overall condition when used in combination with tumor markers and other indicators. In recent years, the CALLY index has been useful in predicting the prognosis of various gastrointestinal cancers, including gastric, esophageal, and colorectal cancers. Supplemental Table 3). The optimal cutoff value for the CALLY index in this study was 4.0, which is higher than the values reported in previous studies (generally 1.7 to 3.5). This difference may be attributed to the selection criteria. All patients in this study had resectable PDAC and underwent upfront R0 resections. Such patients generally have better nutritional reserves and lower inflammatory responses than those with advanced or unresectable cancer, which may have led to an elevated cut-off value. Furthermore, while the 5-year OS was used as the primary ROC endpoint to reflect standard surgical outcomes, we confirmed that the optimal cutoff was 4.0 when the 3-year OS was used as the endpoint, suggesting the stability of this value within our cohort. Nevertheless, whether this cutoff is universal remains to be validated, and further large-scale, multi-institutional studies are required to establish the most appropriate threshold across the different clinical stages of PDAC. Research on the CALLY index for PDAC is limited, and this study provides important evidence to support its usefulness in PDAC.

The prognostic predictive ability of the CALLY index has a significant potential in clinical practice, as demonstrated in this study. Investigation of the preoperative CALLY index may enable the preoperative identification of high-risk patients simply and objectively—patients who cannot be fully captured with conventional staging systems, including the tumor–node–metastasis classification. For example, in patients with a low preoperative CALLY index, it provides valuable information for developing personalized treatment plans, including actively considering the addition of neoadjuvant chemotherapy, selecting more effective postoperative adjuvant therapies, and increasing postoperative surveillance. Future research is expected to clarify how the CALLY index dynamically changes pre- and post-treatment, including surgery or chemotherapy, and how this relates to evaluating treatment effectiveness and long-term outcomes. As more findings accumulate, the CALLY index will become an even more important biomarker for pancreatic cancer diagnosis and treatment.

This study is associated with several limitations. First, this was a single-center retrospective review, and the possibility that unknown biases in the case selection may have affected the results cannot be ruled out. Second, the sample size of 120 cases was relatively small, which potentially lacks sufficient statistical power, particularly for subgroup analyses. Third, the cutoff value (4.0) for the CALLY index, identified using the ROC curve in this study, was specific to our cohort; hence, the optimal cutoff may differ in other patient populations, ethnic groups, or survival periods. Therefore, further validation is warranted to confirm the universality of this cut-off value. Finally, this study spans a long inclusion period, from 2007 to 2019, during which treatment strategies for PDAC have evolved substantially. These temporal changes limit the validity of the analysis of all patients in a single cohort. Furthermore, to overcome these limitations and firmly establish the clinical utility of the CALLY index, a large-scale, prospective, multicenter study will be crucial to validate our findings.

In conclusion, this study revealed that the preoperative CALLY index is an independent prognostic factor for OS in patients undergoing surgery for PDAC. It reflects preoperative systemic inflammatory, nutritional, and immune status. The CALLY index is a useful and convenient tool that complements traditional clinicopathological factors and helps improve risk stratification and personalized treatment strategies for patients with PDAC.

## Electronic Supplementary Material

Below is the link to the electronic supplementary material.

Supplementary Material 1

Supplementary Material 1. Area under the receiver operating characteristic curve to determine the cutoff value of Neutrophil, Lymphocyte, CRP and Albumin levels to predict the 5-year overall survival. CRP C-reactive protein, AUC Area under the curve

Supplementary Material 2. Comparison of the overall survival between the high and low groups for neutrophils, lymphocytes, CRP, and albumin. CRP C-reactive protein

Supplementary Material 2. Comparison of the overall survival between the high and low groups for the CALLY index, SII, PNI, and NLR in CA19-9-positive patients. CALLY index C-reactive protein–albumin–lymphocyte index, SII Systemic inflammation index, PNI Prognostic nutritional index, NLR Neutrophil-to-lymphocyte ratio, MST Median survival time
