# Supplementary material for: Preoperative low C-reactive protein–albumin–lymphocyte (CALLY) index is a poor prognostic indicator for overall survival in patients undergoing surgery for pancreatic ductal adenocarcinoma
Source: Surg Today. 2026 Mar 19;56(8):1595–602. doi: 10.1007/s00595-026-03270-8 (PMC13379495; doi:10.1007/s00595-026-03270-8)
Supplement: Supplementary file 1 — Supplementary Material 1 [file 595_2026_3270_MOESM1_ESM.docx]

**Supplemental Table 1** A univariate analysis of the clinicopathological factors predicting overall survival, including composite indices, in CA19-9-positive patients

| **Variables** | | **Number of patients**  **(n = 68)** | ***p*-value^a^** |
| --- | --- | --- | --- |
| **Age (years)** | ≥70 / <70 | 39 / 39 | 0.880 |
| **Body mass index (kg/m^2^)** | ≥22 / <22 | 28 / 40 | 0.969 |
| **Sex** | Female / Male | 35 / 33 | 0.891 |
| **Obstructive jaundice drainage** | Yes / No | 26 / 42 | 0.480 |
| **Surgical procedure** | TP or PD / DP | 48 / 20 | 0.583 |
| **Postoperative complications** | C–D ≥Ⅲ / C-D <Ⅲ | 19 / 49 | 0.463 |
| **Pathological T factor** | T3T4 / T1T2 | 99 / 21 | 0.631 |
| **Pathological N factor** | N1 / N0 | 27 / 41 | 0.255 |
| **Adjuvant chemotherapy** | No / Yes | 17 / 51 | **0.021** |
| **CEA (ng/mL)** | ≥5 / <5 | 23 / 45 | 0.130 |
| **CALLY index** | **<4.0 / ≥4.0** | **33 / 35** | **0.045** |
| **SII (×10^3^)** | ≥456 / <456 | 34 / 34 | **0.041** |
| **PNI** | <48.7 / ≥48.7 | 47 / 21 | **0.019** |
| **NLR** | ≥2.1 / <2.1 | 38 / 30 | **0.009** |

^a^Log-rank test

*TP* total pancreatectomy, *PD* pancreaticoduodenectomy, *DP* distal pancreatectomy, *C–D* Clavien–Dindo grade, *CA19-9* Carbohydrate antigen 19-9, *CEA* Carcinoembryonic antigen, *CALLY index* C-reactive protein–albumin–lymphocyte index, *SII* Systemic inflammation index, *PNI* Prognostic nutritional index, *NLR* Neutrophil-to-lymphocyte ratio

**Supplemental Table 2** A multivariate analysis of the clinicopathological factors predicting overall survival, including composite indices, in CA19-9-positive patients.

| **Variables** | | **Model 1**  **(with CALLY index)** | | **Model 2**  **(with SII)** | | **Model 3**  **(with PNI)** | | **Model 4**  **(with NLR)** | |
| --- | --- | --- | --- | --- | --- | --- | --- | --- | --- |
|  |  | HR  (95% CI) | p-value^a^ | HR  (95% CI) | p-value^a^ | HR  (95% CI) | p-value^a^ | HR  (95% CI) | p-value^a^ |
| Adjuvant chemotherapy | No / Yes | 1.96  (1.04-3.96) | **0.039** | 1.98  (1.05-3.74) | **0.034** | 1.94  (1.03-3.65) | **0.041** | 2.27  (1.20-4.29) | **0.011** |
| CALLY index | <4.0 / ≥4.0 | 1.71  (0.95-3.08) | 0.073 |  |  |  |  |  |  |
| SII (×10^3^) | ≥456 / <456 |  |  | 1.76  (0.97-3.17) | 0.060 |  |  |  |  |
| PNI | <48.7 / ≥48.7 |  |  |  |  | 2.09  (1.06-4.14) | **0.034** |  |  |
| NLR | ≥2.1 / <2.1 |  |  |  |  |  |  | 2.29  (1.28-4.11) | **0.006** |

^a^Cox Regression Analysis

*HR* hazard ratio, *CI* confidence interval*, CALLY index* C-reactive protein–albumin–lymphocyte index, *SII* Systemic inflammation index, *PNI* Prognostic nutritional index, *NLR* Neutrophil-to-lymphocyte ratio

**Supplemental Table 3** Summary of the published studies on the prognostic value of the CALLY Index for overall survival after surgical resection of gastrointestinal cancer

| **Number** | **Author**  **(year), [reference]** | **Cancer type** | **Cutoff value** | **Percentage below the**  **Cutoff value** | **Hazard ratio**  **(95% CI)** | **Multivariate factors:**  **Including**  **tumor markers** | **Multivariate factors:**  **Including composite index** |
| --- | --- | --- | --- | --- | --- | --- | --- |
| 1 | Kawahara  (2024), [6] | Pancreatic cancer | 1.9 | 31% | 1.772  (1.362-2.305) | CA19-9 | None |
| 2 | Tsunematsu  (2022), [11] | Cholangiocarcinoma | 3.5 | 61% | 2.07  (1.11-3.89) | CA19-9 | NLR, PLR, CAR |
| 3 | Iida  (2021), [12] | Hepatocellular carcinoma | 5 | 39% | 1.81  (1.21-2.71)^a^ | AFP, PIVKA-II | None |
| 4 | Takeda  (2024), [13] | Colorectal cancer | 2 | 30% | 2.79  (1.32-5.92) | CA19-9, CEA | NLR, PLR |
| 5 | Aoyama  (2024), [14] | Esophageal cancer | 5 | 51% | 2.310  (1.416-3.767) | None | None |
| 6 | Ma  (2024), [15] | Esophageal cancer | 2.4 | 30% | 3.86  (2.03-7.34) | None | None |
| 7 | Feng  (2024), [16] | Esophageal cancer | 1.7 | 46% | 2.72  (1.98-3.73)^b^ | None | None |
| 8 | Nakashima  (2024), [17] | Gastric cancer | 6.96 | 49% | 3.00  (1.31-6.93) | CA19-9, CEA | NLR, PLR |
| 9 | Fukushima  (2024), [18] | Gastric cancer | 2 | 18% | 2.02  (1.18-3.46) | CEA | PNI |
| 10 | Hashimoto  (2024), [19] | Gastric cancer | 3.28 | 21% | 1.961  (1.19-3.23) | None | None |
| 11 | Okugawa  (2024), [20] | Gastric cancer | 4.93 | 54% | 2.57  (1.62-4.07) | None | None |
| 12 | Aoyama  (2024), [21] | Gastric cancer | 5 | 32% | 1.791  (1.067-3.009) | None | None |
| 13 | Sakurai  (2024), [22] | Gastric cancer | 1.19 | 52% | 1.82  (1.24-2.69) | None | NL PLR, mGPS |
| 14 | Toda  (2025), [23] | Gastric cancer | 2 | 14% | 2.11  (1.22-3.63) | None | None |
| **Our study** | **Matsumoto (2025)** | **Pancreatic ductal adenocarcinoma** | **4.0** | **47%** | **2.33**  **(1.39-3.59)** | **CA19-9, CEA** | **SII, PNI, NLR** |

*CI* Confidence interval, *CEA* Carcinoembryonic antigen, *CA 19-9* Carbohydrate antigen 19-9, *AFP* Alpha-fetoprotein, *PIVKA-II* Protein induced by vitamin K absence or antagonist-II, *NLR* Neutrophil-to-lymphocyte ratio, *PLR* Platelet-to-lymphocyte ratio,

*CAR* C-reactive protein-albumin ratio, *PNI* Prognostic nutritional index, *mGPS* modified Glasgow prognostic score, *SII* Systemic inflammation index
