# Supplementary figures and images for: Preoperative low C-reactive protein–albumin–lymphocyte (CALLY) index is a poor prognostic indicator for overall survival in patients undergoing surgery for pancreatic ductal adenocarcinoma
Source: Surg Today. 2026 Mar 19;56(8):1595–602. doi: 10.1007/s00595-026-03270-8 (PMC13379495; doi:10.1007/s00595-026-03270-8)

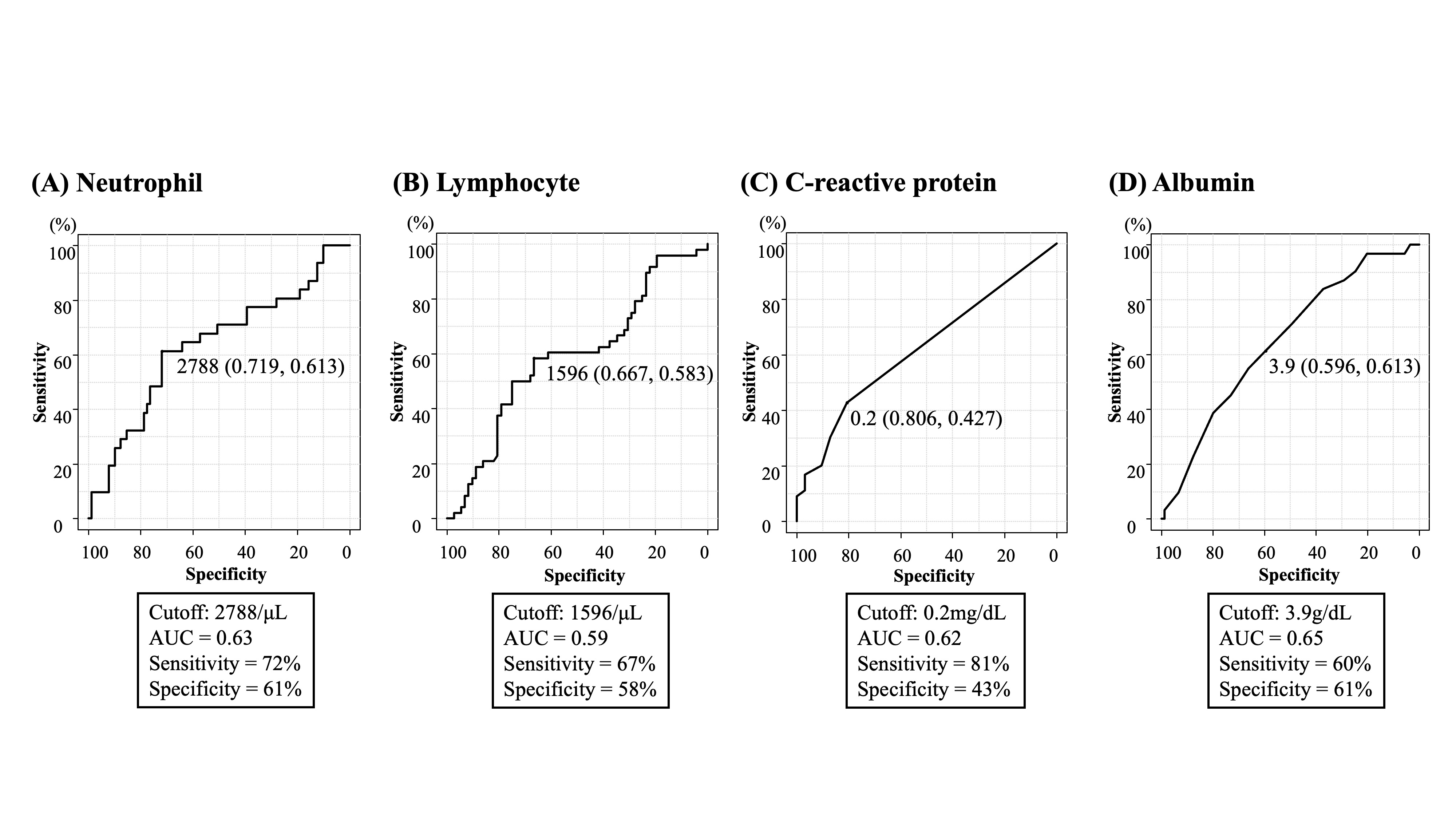

Supplement: Supplementary file 2 — Supplementary Material 1. Area under the receiver operating characteristic curve to determine the cutoff value of Neutrophil, Lymphocyte, CRP and Albumin levels to predict the 5-year overall survival. CRP C-reactive protein, AUC Area under the curve [file 595_2026_3270_MOESM2_ESM.jpg]

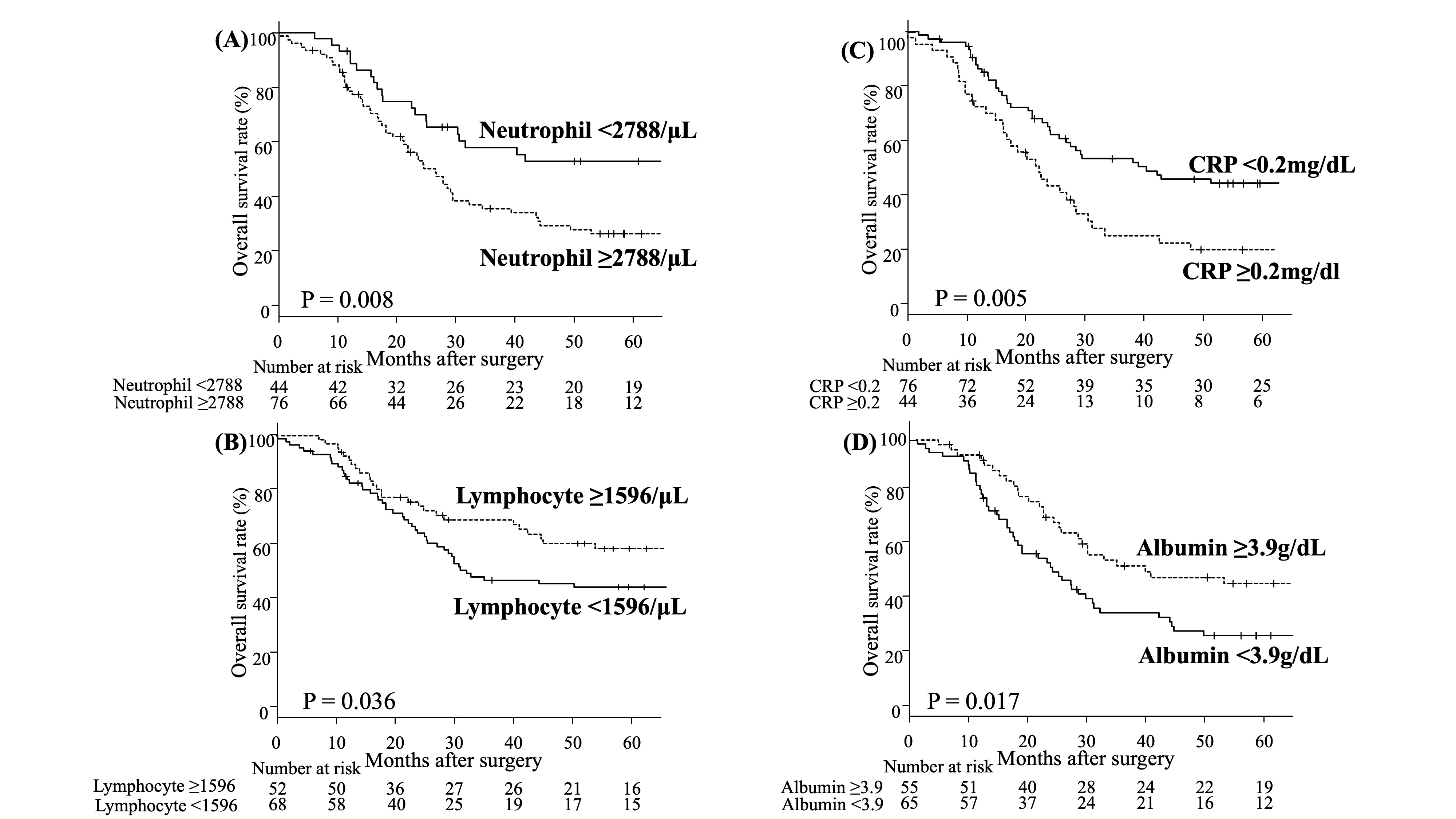

Supplement: Supplementary file 3 — Supplementary Material 2. Comparison of the overall survival between the high and low groups for neutrophils, lymphocytes, CRP, and albumin. CRP C-reactive protein [file 595_2026_3270_MOESM3_ESM.jpg]

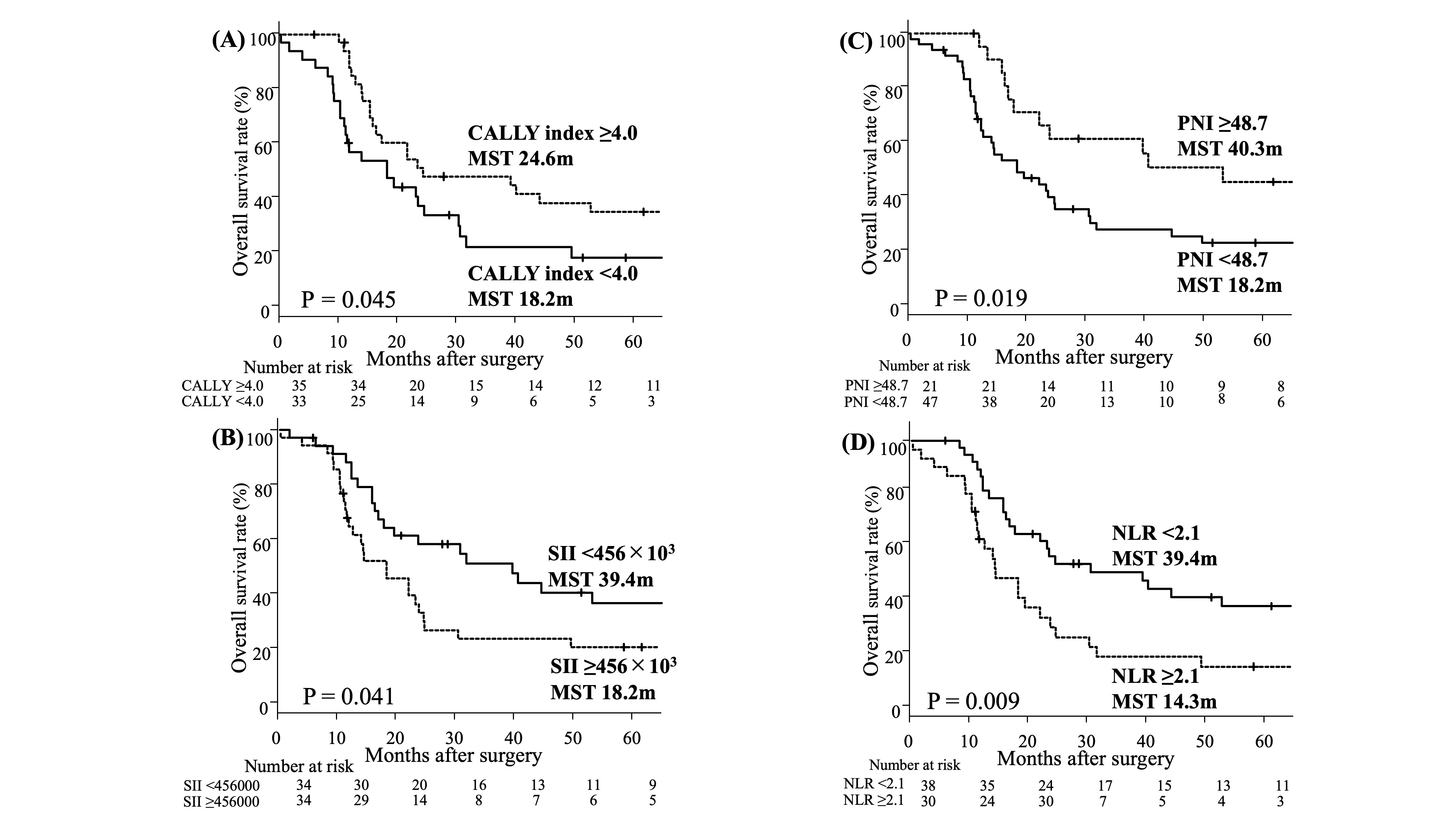

Supplement: Supplementary file 4 — Supplementary Material 2. Comparison of the overall survival between the high and low groups for the CALLY index, SII, PNI, and NLR in CA19-9-positive patients. CALLY index C-reactive protein–albumin–lymphocyte index, SII Systemic inflammation index, PNI Prognostic nutritional index, NLR Neutrophil-to-lymphocyte ratio, MST Median survival time [file 595_2026_3270_MOESM4_ESM.jpg]
